# Clusters of Pregnant Women with Severe Acute Respiratory Syndrome Due to COVID-19: An Unsupervised Learning Approach

**DOI:** 10.3390/ijerph192013522

**Published:** 2022-10-19

**Authors:** Isadora Celine Rodrigues Carneiro, Sofia Galvão Feronato, Guilherme Ferreira Silveira, Alexandre Dias Porto Chiavegatto Filho, Hellen Geremias dos Santos

**Affiliations:** 1 Instituto Carlos Chagas, Fundação Oswaldo Cruz, Curitiba 81310-020, Brazil; 2 Faculdade de Saúde Pública, Universidade de São Paulo, São Paulo 01246-904, Brazil

**Keywords:** COVID-19, severe acute respiratory syndrome, pregnant women, hospitalizations, healthcare systems and management

## Abstract

COVID-19 has been widely explored in relation to its symptoms, outcomes, and risk profiles for the severe form of the disease. Our aim was to identify clusters of pregnant and postpartum women with severe acute respiratory syndrome (SARS) due to COVID-19 by analyzing data available in the Influenza Epidemiological Surveillance Information System of Brazil (SIVEP-Gripe) between March 2020 and August 2021. The study’s population comprised 16,409 women aged between 10 and 49 years old. Multiple correspondence analyses were performed to summarize information from 28 variables related to symptoms, comorbidities, and hospital characteristics into a set of continuous principal components (PCs). The population was segmented into three clusters based on an agglomerative hierarchical cluster analysis applied to the first 10 PCs. Cluster 1 had a higher frequency of younger women without comorbidities and with flu-like symptoms; cluster 2 was represented by women who reported mainly ageusia and anosmia; cluster 3 grouped older women with the highest frequencies of comorbidities and poor outcomes. The defined clusters revealed different levels of disease severity, which can contribute to the initial risk assessment of the patient, assisting the referral of these women to health services with an appropriate level of complexity.

## 1. Introduction

Since its emergence as a pandemic in 2020, COVID-19 has been widely explored in relation to its epidemiological characteristics, symptoms, and outcomes, as well as comorbidities and risk factors that may predispose an individual to a severe condition. In the general population, approximately 80% of cases are asymptomatic or mild, with flu-like symptoms, such as fever, myalgia, headache, malaise, cough, and sore throat [1]. Of the remaining cases, 15% are severe and 5% critical, generally requiring supplemental oxygen and mechanical ventilation, respectively [2]. Nevertheless, there are groups at greater risk of developing the severe form of the disease, such as elderly individuals, individuals with comorbidities, and pregnant women [3,4].

The pregnancy-puerperal period deserves special attention as it involves immunophysiological, hormonal, and cardiopulmonary changes that can predispose an individual to complications resulting from respiratory infections such as COVID-19 [5,6]. Similarly to the general population, pregnant women are also frequently asymptomatic [7]. However, some studies have reported more severe cases of COVID-19 in the third trimester of pregnancy [7,8], possibly due to the greater overload of the cardiopulmonary system observed at the end of pregnancy, as well as among pregnant women who have comorbidities [7,9,10] or an unfavorable socioeconomic condition [11], which could result in difficulty in accessing quality health services in a timely manner.

Severe morbidity and mortality effects from COVID-19 are directly related to the evolution of the disease from a flu-like condition to a severe acute respiratory syndrome [12], which can result in poor outcomes for mothers and their babies, such as emergency cesarean section procedures and preterm birth [13,14]. Brazil is the country with the highest rates of maternal mortality from COVID-19 [6]. According to the Observatório Obstétrico Brasileiro (OOBr), maternal deaths from COVID-19 in the first half of 2021 (911 deaths until 31 May) exceeded the number recorded in the entire year of 2020 (544 deaths) [15]. Delays in initial care and hospitalization [16] and barriers to accessing resources available in specialized intensive care services, such as mechanical ventilators [11], were frequently experienced by this group and may be the main reason for the fatal outcome.

Unsupervised learning approaches are used when several characteristics are observed for each individual, but there is no previously defined outcome of interest, responsible for guiding the analysis. Therefore, the main objective is to understand relationships between variables or observations and to simplify or reduce data or identify target groups to which specific actions can be proposed [17]. Principal component analysis, multiple correspondence analysis, and cluster analysis are some examples of techniques that may be used for these purposes.

Specifically regarding COVID-19 research, these approaches were applied to reveal profiles of disease severity in the general population based on symptoms, laboratory measures, and comorbidities [18,19,20,21,22] and to detect vaccine hesitancy segments using data on intentions to take the COVID-19 vaccine, beliefs about the vaccine and the disease, and adherence to non-pharmacological interventions [23]. In this setting, identifying clusters of pregnant and postpartum women with different risk profiles for the severe form of COVID-19 can collaborate to the proposal of preventive measures, guidelines for seeking care in services of different levels of complexity, and for the organization of specific care for this population. This study aimed to identify clusters of pregnant or postpartum women with SARS resulting from COVID-19, according to symptoms, comorbidities, and hospital characteristics, using data routinely generated by healthcare services during the COVID-19 pandemic in Brazil.

## 2. Materials and Methods

### 2.1. Study Design and Participants

A nationwide cross-sectional study was carried out using an analysis of public data from the Influenza Epidemiological Surveillance Information System (Sistema de Informação da Vigilância Epidemiológica da Gripe, SIVEP-Gripe) from March 2020 to August 2021. The SIVEP-Gripe is the official Brazilian system for recording cases and deaths of Severe Acute Respiratory Syndrome (SARS). It is coordinated by the Ministry of Health since the Influenza A (H1N1) pandemic, which occurred in 2009. This information system started recording hospitalized SARS cases resulting from COVID-19 in February 2020. For the present study, we considered women aged between 10 and 49 years old (fertile period), whose gestational stage was classified as the first (n=1092), second (n=3276), or third (n=7455) trimester of pregnancy; pregnant women whose gestational age was not informed (n=587); and those who provided affirmative answers for the field corresponding to the puerperium—referring to women whose childbirth occurred at most 42 days earlier (n=3999), resulting in a total of 16,409 women.

### 2.2. Study Variables

The following variables were selected from the SIVEP-Gripe:

1. **Demographic and obstetric characteristics:** maternal age group (10–17, 18–34, 35–42, and 43–49); race/skin color (White, mixed, Black, Asian, and Indigenous); gestational stage (first—1st; second—2nd; and third—3rd trimester, puerperium, or no available data on gestational age). We dichotomized race/skin color into White/Asian and Indigenous/mixed/Black/no available (NA) data;
2. **Symptoms (yes or no):** 1: Ageusia; 2: anosmia; 3: abdominal pain; 4: diarrhea; 5: vomiting; 6: dyspnea; 7: respiratory distress; 8: oxygen saturation less than 95%; 9: chest pain; 10: fever; 11: cough; 12: sore throat; 13: fatigue; 14: myalgia; 15: inappetence; 16: headache; 17: malaise; 18: nasal congestion;
3. **Comorbidities (yes or no):** 19: Chronic heart disease; 20: asthma; 21: diabetes mellitus; 22: hypertension; 23: immunodeficiency or immunodepression; 24: obesity; 25: eclampsia;
4. **Hospital characteristics:** Use of mechanical ventilation (MV) (26a: yes, invasive MV; 26b: yes, non-invasive MV, or no); 27: admission to the intensive care unit (ICU) (yes or no); 28: hospital outcome (discharge or death).

The SIVEP-Gripe form has an open field for the description of other symptoms and comorbidities. We analyzed these fields to retrieve symptoms and comorbidities not selected in the pre-defined corresponding fields, and we assessed characteristics frequently described for the pregnant and postpartum women population but that had no pre-defined field in this form (inappetence, nasal congestion, headache, chest pain, myalgia, malaise, and preeclampsia/eclampsia). With the exception of the use of MV, symptoms, comorbidities, and hospital characteristics were transformed into dichotomous variables (yes or non-occurrence). Furthermore, since in most cases the variable was filled in only when the characteristic was observed, empty fields were grouped into the non-occurrence category.

### 2.3. Statistical Analysis

Multiple correspondence and agglomerative hierarchical clustering analyses were performed to identify segments within the pregnant and postpartum women population (Figure S1). These exploratory approaches together can provide relevant information for the clinical management of each specific subgroup in order to make better use of available resources.

As our first step, we applied multiple correspondence analyses (MCA) on categorical variables represented by symptoms, comorbidities, and hospital characteristics as a preprocess procedure to derive a set of continuous and uncorrelated principal components (PCs) [24]. This method makes it possible to summarize the information contained in categorical variables into a lower-dimensional space, which explains the maximum amount of data variability [25]. The MCA interpretation is based on the estimated eigenvalues and eigenvectors. The former are positive quantities related to inertia, which represents the total amount of explained variability (percentage of inertia) by a given PC, while the latter represents the PC orientation [24]. The eigenvalues order the PCs from the one that most explains the variability of the data to the one that explains the least. PCs totaling 50% of explained variability were retained for Agglomerative Hierarchical Cluster Analysis.

Cluster analysis includes a set of methods suitable for exploring data sets aimed at segmenting a heterogeneous population into clusters of individuals who present a high degree of homogeneity regarding the studied variables, as well as a high degree of heterogeneity between the defined clusters. These methods are divided into hierarchical and partitioning procedures, the former being of great practical interest since it does not require a prior definition of the number of clusters that has to be obtained. Hierarchical procedures can be further subdivided into agglomerative and divisive according to whether the initial step of the clustering algorithm is related to each individual being considered as a cluster or whether all individuals are a single cluster, respectively [26].

There are many agglomerative hierarchical clustering procedures, but all of them can be described according to the following basic steps: first, we have *n* clusters, each containing a single *p*-dimensional entity (individual) and an n×n symmetric matrix of distances. The task consists of searching for the most similar pair of clusters that will be merged; next, the distance matrix entries are updated, and this task is repeated until all individuals are in a single cluster; finally, we can display the procedure’s results in a hierarchical tree and choose where to cross-section it to define the most suitable number of clusters [27].

We identified segments within the pregnant and postpartum women population by applying hierarchical clustering on the retained PCs in an agglomerative manner, using Ward’s algorithm and Euclidean distance. This approach is based on joining clusters for which its combination implies the minimum loss of information, i.e., the smallest increase in an error sum of squares criterion. Thus, at each algorithm step, all possible pairs of clusters are evaluated, and pairs with a combination that results in a minimum loss of information are joined [27]. The number of clusters was determined by the visual inspection of the hierarchical tree; within-cluster homogeneity increases, represented by an inertia plot; and cluster interpretability [24]. The defined clusters were presented on a map formed by the first two PCs.

To validate and interpret these clusters, we estimated the absolute and relative frequencies of symptoms and comorbidities, as well as hospital, demographic, and obstetric characteristics. Furthermore, to assess whether clusters had different COVID-19 severity profiles, we compared these characteristics between the clusters by estimating relative risks (RR) and corresponding 95% confidence intervals (95% CI). Analyses were performed with R software version 4.1.3 by using the ‘ca’ and ‘FactoMineR’ packages.

## 3. Results

### 3.1. Multiple Correspondence Analysis

Between March 2020 and August 2021, 16,409 pregnant or postpartum women were registered on SIVEP-Gripe with SARS due to COVID-19. We retained 10 PCs from the MCA applied to 28 categorical variables related to symptoms, comorbidities, and hospital characteristics. These PCs summarized 51.18% of the total percentage of inertia (Table 1).

**Figure 1:**
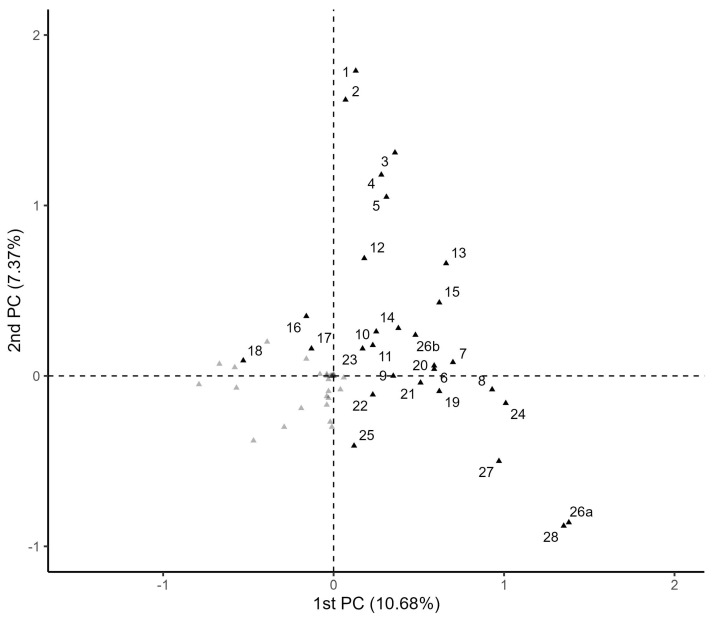
Multiple correspondence analysis results for the first and the second principal component (PC). The black dots are “yes” categories, numbered according to the variable labels presented in the methods section. Gray dots correspond to the “non–occurrence” category for symptoms, comorbidities, or hospital characteristics.

Overall, the first PC distinguished women with mild symptoms (positive responses to 16: headache; 17: malaise; and 18: nasal congestion), related to a flu-like condition, and without comorbidities (upper left quadrant) from those symptomatic women with comorbidities, whereas the second PC seems to differentiate moderate from severe cases, the former represented by women with positive responses for ageusia (1), anosmia (2), abdominal pain (3), diarrhea (4), and vomiting (5) (upper right quadrant) and the latter by poor hospital outcomes (26a: invasive MV; 27: ICU admission and 28: death, in the lower right quadrant) (Figure 1).

### 3.2. Agglomerative Hierarchical Clustering

**Figure 2:**
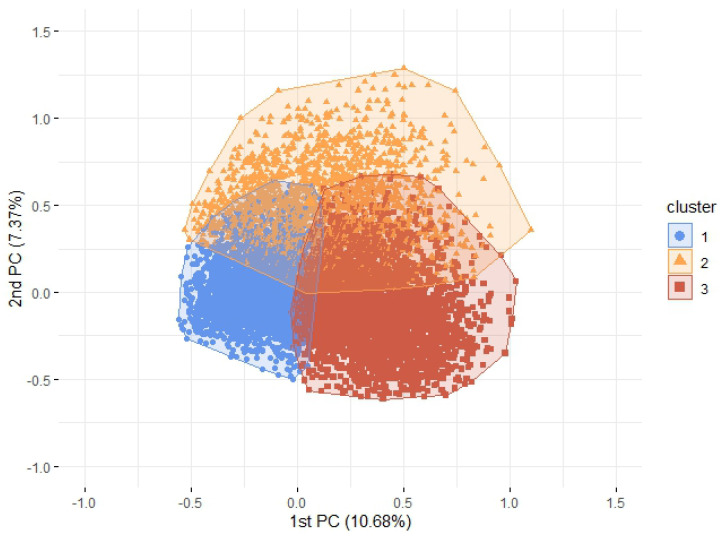
Distribution of the pregnant and postpartum women with SARS due to COVID-19 into the three clusters according to the first and the second principal component (PC).

Hierarchical clustering was applied to the top 10 PCs retained from the MCA. A visual inspection of the hierarchical tree (Figure S2) and inertia plot (Figure S3) suggested a population segmentation into three clusters (Figure 2).

In Table 2, we present the frequency of affirmative answers for the analyzed variables according to clusters. Cluster 1 (n=7866; 48%) grouped younger women with the lowest frequencies of comorbidities and poor hospital outcomes (i.e., MV, ICU admission, and death), while cluster 3 (n=6327; 38.5%) aggregated women with the highest frequencies for these characteristics, indicating segments of mild and severe disease, respectively. Cluster 2 (n=2216; 13.5%), on the other hand, presented higher frequencies of anosmia and ageusia, as well as gastrointestinal symptoms. Additionally, this group is situated between cluster 1 and cluster 3 regarding positive answers for symptoms, comorbidities, and poor hospital outcomes, suggesting a segment of patients with moderate cases of the disease.

Table 3 shows the comparison of symptoms, comorbidities, hospital, demographic, and obstetric characteristics frequencies between clusters, considering cluster 1 as the reference group.

It shows a gradual increase in frequencies from the first to the third cluster for variables related to hospital characteristics, lower airway respiratory symptoms (except fatigue), and comorbidities (except eclampsia). Furthermore, cluster 2 presented the highest relative risks for anosmia, ageusia, and gastrointestinal symptoms. Flu-like symptoms presented the most similar frequencies between the clusters (RR close to 1). In addition, cluster 1, despite being the one with a mild risk profile, had a higher frequency of pregnant women who were Indigenous, mixed, Black, or without race/skin color information and aged less than 35 years old, suggesting a group with unfavorable socioeconomic condition.

## 4. Discussion

In this study, we analyzed data from pregnant and postpartum women with SARS due to COVID-19 in Brazilian health services between March 2021 and August 2021. We identified three clusters in this population, corresponding to different disease severity profiles. In summary, cluster 1 was represented by younger women, with the lowest frequencies of comorbidities and poor hospital outcomes. This group presented flu-like symptoms, suggesting a segment of mild COVID-19 cases. Cluster 2 was represented by women who reported a variety of symptoms, mainly nasopharyngeal (ageusia and anosmia) and gastrointestinal. Finally, cluster 3 showed a more severe profile of COVID-19, grouping older women with the highest frequencies of comorbidities, mainly obesity, who most often required invasive MV and ICU admission. Mortality was also higher in this segment of the population.

Women in the pregnancy-puerperal period are routinely followed up by specialized health services, both during prenatal care and at the time of childbirth. However, given the diagnosis of viral infections, especially those that affect the airways, this population needs priority care, due to the implications of the disease for women and their babies. In Brazil, the COVID-19 pandemic highlighted pregnant women’s difficulties in accessing health services, which, consequently, led to a delay in providing care to this population, which may have contributed to the high rate of maternal mortality from COVID-19 [6,16].

The population segmentation that we performed identified a group (cluster 1) with a higher frequency of younger women of Indigenous, mixed, Black, or not available race/skin color information who, despite having symptoms consistent with a mild disease, required care at health facilities. This group corroborates the expected profile for most cases of COVID-19, representing a segment of pregnant/postpartum women with symptoms similar to a flu-like condition. A systematic review [28] highlighted that the course of COVID-19 in pregnant women is similar to that of the general population, with most cases being asymptomatic and those with symptoms presenting a mild condition. Dashraath et al. (2021) [29] also reported no differences between pregnant and non-pregnant women regarding symptoms of COVID-19, with a predominance of fever, cough, dyspnea, and lymphopenia. Importantly, as these symptoms are commonly seen in colds and flu, this profile can confuse the diagnosis of COVID-19 and delay proper management. Additionally, the condition of social vulnerability requires close monitoring, as it is indicative of greater difficulty in accessing healthcare services.

Cluster 2 aggregated women who most frequently reported gastrointestinal symptoms, ageusia, and anosmia. Teixeira et al. (2021) [30] reported ageusia and anosmia as the most common clinical symptoms of COVID-19 in pregnant women with comorbidities in a study carried out in Rio de Janeiro. These are the most specific symptoms in COVID-19 infections, with great potential to be used as “flag symptoms” in the context of initial risk assessments in health facilities [31]. Multiawati et al. (2021) [32] performed a meta-analysis aiming to estimate the prevalence of anosmia and dysgeusia in patients with COVID-19 and concluded that these symptoms are more prevalent in patients with COVID-19 than in those with other respiratory diseases.

On the other hand, gastrointestinal symptoms are also common in several viral infections and represent a warning sign for the worsening of the general condition of patients, especially as a result of dehydration. A review study focusing on the gastrointestinal symptoms of COVID-19 [31] concluded that, similarly to the general population, pregnant women may experience gastrointestinal manifestations such as diarrhea, abdominal pain, nausea, and a loss of appetite. One hypothesis is that SARS-CoV-2 binds to human ACE-2 receptors that are also present in intestinal cells, hepatocytes, and cholangiocytes, making the gastrointestinal tract a potential route for infection. Thus, pregnant women with gastrointestinal symptoms should be closely monitored in appropriate health facilities, as these signs can indicate both SARS-CoV-2 and other infections and can also lead to secondary conditions such as dehydration.

Studies that analyzed data from SIVEP-Gripe to describe characteristics related to maternal morbidity and mortality due to SARS from COVID-19 infection highlighted characteristics similar to those of the cluster 3 profile [33,34]. Takemoto et al. (2020) [33] described a fatality rate of 12.7% among 978 pregnant and postpartum women with SARS from COVID-19 until 18 June 2020. Among the fatal cases, the authors highlighted the frequency of at least one comorbidity (48.4%), as well as ICU admission (58.9%) and invasive MV (53.2%). Additionally, the main risk factors for maternal death were being postpartum at the onset of SARS, being obese, and having diabetes or cardiovascular disease. Menezes et al. (2020) [34] analyzed data up to 14 July 2020 and identified 2475 pregnant and postpartum women with SARS due to COVID-19 and 204 deaths. The authors emphasized that age over 35 years, obesity, diabetes, black skin color, living in a peri-urban area, not having access to family health strategy, or living more than 100 km from the reporting hospital were risk factors for adverse outcomes (death, ICU admission, and MV).

According to our analysis, in cluster 3, older women in the puerperium period and with comorbidities were more frequent. This cluster also had a higher frequency of invasive MV, ICU admission, and death. In addition, symptoms indicative of more severe cases of the disease, such as dyspnea, fatigue, oxygen saturation less than 95%, and respiratory distress, were also more prevalent in this group. Lassi et al. (2021) [35] systematically reviewed studies comparing pregnant women with severe and non-severe COVID-19 and highlighted an increased risk of severe COVID-19 cases for older pregnant women (over 35 years old) with comorbidities (obesity, diabetes, and preeclampsia). In addition, the authors reported a high risk of adverse neonatal outcomes, such as preterm birth, from women with a severe case of the disease.

Among the comorbidities analyzed in our study, obesity was the most frequently observed in cluster 3. In the general population, this condition has been recognized as one of the main risk factors for severe COVID-19 cases [3]. Additionally, among pregnant women, obesity may also imply a higher risk of cesarean section [36]. According to a study review of pregnant women with COVID-19, obesity doubled the risk of death [37]. Furthermore, a multicenter cohort study of pregnant women with and without a diagnosis of COVID-19 [13] revealed a higher frequency of overweight in early pregnancy, as well as preeclampsia/eclampsia, ICU admission, maternal mortality, and preterm birth among pregnant women with diagnoses of COVID-19. These results suggest that the presence of comorbidities, especially obesity, increases the risk of a worse prognosis of COVID-19.

Finally, it is important to emphasize that, in addition to the direct impacts of the COVID-19 pandemic, indirect consequences related to delays in obstetric care due to suspensions of consultations or changes in birth plans, as well as worsening socioeconomic status and increased mental suffering due to fear of illness, implied reduced access to and quality of obstetric care during the pregnancy-puerperal period, thus favoring poor outcomes [38,39].

Our study has limitations that need to be mentioned. First, we analyzed a public database, so the number of cases we identified may be underestimated due to the underreporting of cases, for example, from patients who had symptoms and did not undergo tests or were misdiagnosed. Another limitation refers to the absence of data on neonatal outcomes in this dataset. Finally, we considered data from the beginning of the pandemic to August 2021, since we chose to analyze a setting at the moment immediately before the start of vaccination of pregnant women (July 2021).

Despite the advances achieved in maternal and child health during the last decades, with the adoption of public policies aimed at monitoring prenatal care and childbirth, there are still several barriers to be faced for access to quality health services in Brazil. The alarming number of maternal deaths from COVID-19 points to delays in health care access for this population that are exacerbated by socioeconomic and spatial inequalities [34,40,41].

## 5. Conclusions

Our results highlighted the importance of continuously monitoring the population of pregnant and postpartum women, as well as of properly referring them to specialized care services prepared to attend this specific population. The clusters identified in this study can contribute to the initial assessment of the patient, differentiating general conditions from those most likely to be related to COVID-19, and those indicative of severe cases, assisting in the referral of these women to health services with the appropriate level of complexity. Moreover, different profiles reinforce key variables that could be used in the initial risk assessment and in the prognostic evaluation, helping to improve the clinical management of each specific subgroup and, thus, implying the more efficient use of scarce resources currently available in healthcare services.

## Figures and Tables

**Table 1:** Percentage of inertia for the first 10 principal components.

Principal Components	Eigenvalue	Percentage of Inertia	Cumulative Percentage of Inertia
1	0.110	10.681	10.681
2	0.076	7.368	18.049
3	0.054	5.259	23.309
4	0.048	4.675	27.984
5	0.044	4.326	32.310
6	0.044	4.265	36.575
7	0.040	3.950	40.526
8	0.037	3.604	44.130
9	0.036	3.545	47.675
10	0.036	3.499	51.175

**Table 2:** Relative frequency of symptoms, comorbidities, hospital, demographic, and obstetric characteristics per cluster and in the total population of pregnant or postpartum women with SARS due to COVID-19.

Variables (Category = Yes)	Cluster 1 (*n* = 7866)	Cluster 2 (*n* = 2216)	Cluster 3 (*n* = 6327)	Total (*n* = 16,409)
**Hospital characteristics**				
Death	1.58	5.82	23.52	10.61
ICU admission	7.70	19.58	57.26	28.42
Invasive MV	1.27	7.99	31.25	13.74
Non-invasive MV	15.34	39.76	54.39	33.69
**Lower respiratory symptoms**				
Oxygen saturation less than 95%	6.79	38.63	77.64	38.41
Respiratory distress	19.48	50.81	74.35	44.87
Dyspnea	29.21	63.09	89.98	57.22
Fatigue	9.71	43.23	30.47	22.24
**Gastrointestinal symptoms**				
Abdominal pain	4.73	16.43	5.83	6.73
Vomiting	8.56	19.04	9.80	10.45
Diarrhea	7.56	20.49	8.60	9.71
Inappetence	0.57	1.22	1.83	1.15
**Nasopharyngeal symptoms**	1.88	87.68	1.15	13.19
Anosmia	4.64	92.87	2.61	15.77
Sore throat	18.04	32.76	18.60	20.24
**Flu-like symptoms**				
Nasal congestion	12.97	10.29	4.90	9.49
Malaise	2.03	1.99	1.50	1.82
Headache	19.62	25.36	13.58	18.06
Chest pain	1.98	2.75	2.72	2.37
Cough	57.14	76.13	77.46	67.54
Fever	45.03	63.40	60.90	53.63
Myalgia	4.64	7.76	8.38	6.50
**Comorbidities**				
Obesity	2.36	8.62	12.15	6.98
Chronic heart disease	3.83	7.13	8.98	6.26
Asthma	2.29	3.88	4.87	3.50
Diabetes mellitus	5.35	8.75	10.61	7.84
Systemic arterial hypertension	3.13	4.20	4.08	3.64
Immunodeficiency or immunodepression	1.30	1.49	1.58	1.43
Eclampsia	1.33	0.81	1.61	1.37
**Demographic and obstetric characteristics**				
Age 10–17	5.29	2.48	2.09	3.67
Age 18–34	71.26	69.86	64.36	68.41
Age 35–42	0.21	24.68	29.84	25.06
Age 43–49	0.02	2.98	3.71	2.86
1st trimester	7.08	7.36	5.88	6.65
2nd trimester	15.68	23.19	24.17	19.96
3rd trimester	49.28	49.46	39.24	45.43
Puerperium	23.56	17.24	27.88	24.37
White/Asian	31.30	39.53	38.64	35.24
Indigenous/Pardo/Black or NA data	68.70	60.47	61.36	64.76

**Table 3:** Relative Risk (RR) with 95% confidence interval (CI) for the comparison of symptoms, comorbidities, and hospital characteristics between clusters (cluster 1: reference group) of pregnant and postpartum women with SARS due to COVID-19.

Variables (Category = yes)	Cluster 2 RR (CI95%)	Cluster 3 RR (CI95%)
**Hospital characteristics**		
Death	3.69 (2.90; 4.70)	14.92 (12.46; 17.86)
ICU admission	2.54 (2.27; 2.85)	7.43 (6.87; 8.05)
Invasive/Non-invasive Mechanical Ventilation *	2.87 (2.69; 3.07)	5.15 (4.90; 5.42)
**Lower respiratory symptoms**		
Oxygen saturation less than 95%	5.69 (5.16; 6.27)	11.44 (10.53; 12.43)
Respiratory distress	2.61 (2.46; 2.77)	3.82 (3.64; 4.00)
Dyspnea	2.16 (2.06; 2.26)	3.08 (2.97; 3.19)
Fatigue	4.45 (4.10; 4.83)	3.14 (2.90; 3.39)
**Gastrointestinal symptoms**		
Abdominal pain	3.47 (3.03; 3.98)	1.23 (1.07; 1.42)
Vomiting	2.23 (1.99; 2.49)	1.15 (1.03; 1.27)
Diarrhea	2.71 (2.42; 3.03)	1.14 (1.02; 1.27)
Inappetence	2.13 (1.32; 3.42)	3.20 (2.28; 4.51)
**Nasopharyngeal symptoms**		
Ageusia	46.6 (39.70; 54.71)	0.61 (0.46; 0.81)
Anosmia	20.01 (18.09; 22.14)	0.56 (0.47; 0.67)
Sore throat	1.82 (1.68; 1.96)	1.03 (0.96; 1.11)
**Flu-like symptoms**		
Nasal congestion	0.79 (0.69; 0.91)	0.38 (0.33; 0.43)
Malaise	0.98 (0.70; 1.36)	0.74 (0.57; 0.95)
Headache	1.29 (1.19; 1.41)	0.69 (0.64; 0.75)
Chest pain	1.39 (1.04; 1.86)	1.37 (1.11; 1.70)
Cough	1.33 (1.29; 1.37)	1.36 (1.32; 1.39)
Fever	1.41 (1.35; 1.47)	1.35 (1.31; 1.40)
Myalgia	1.67 (1.40; 1.99)	1.81 (1.59; 2.05)
**Comorbidities**		
Obesity	3.65 (3.00; 4.44)	5.14 (4.39; 6.01)
Chronic heart disease	1.86 (1.55; 2.25)	2.35 (2.05; 2.69)
Asthma	1.70 (1.32; 2.18)	2.13 (1.78; 2.55)
Diabetes mellitus	1.64 (1.39; 1.93)	1.98 (1.76; 2.23)
Systemic arterial hypertension	1.34 (1.06; 1.70)	1.30 (1.10; 1.55)
Immunodeficiency or immunodepression	1.15 (0.78; 1.70)	1.22 (0.93; 1.60)
Eclampsia	0.61 (0.37; 1.00)	1.21 (0.92; 1.58)
**Demographic and obstetric characteristics**		
Age greater than or equal to 35 *	1.18 (1.09; 1.28)	1.43 (1.36; 1.51)
Puerperium *	0.72 (0.65; 0.79)	1.16 (1.10; 1.23)
Indigenous/Pardo/Black or NA data	0.88 (0.85; 0.91)	0.89 (0.87; 0.92)

* The variables mechanical ventilation, maternal age, and gestational stage were dichotomized.

## Data Availability

We analyzed publicly available data, available for download on https://opendatasus.saude.gov.br/dataset/srag-2021-e-2022, accessed on 15 August 2021. We made our R code available on the GitHub repository https://github.com/Hellengeremias/MCA_cluster_analysis.

## Supplementary Materials

The following supporting information can be downloaded at: https://www.mdpi.com/article/10.3390/ijerph192013522/s1. Figure S1: Flowchart of the Multiple Correspondence Analysis-Agglomerative Hierarchical Clustering procedure. Figure S2: Agglomerative Hierarchical Clustering of the pregnant and postpartum women with SARS due to COVID-19 on the top 10 Principal Components. Population was segmented into three clusters. Hierarchical tree (cut off value: 0.04). Figure S3: Inertia plot. Since the increase in homogeneity within the cluster from three to four clusters is small when compared to that observed from two to three clusters, the population was segmented into three clusters.

## Abbreviations

The following abbreviations are used in this manuscript:

SARS	Severe acute respiratory syndrome
SIVEP-Gripe	Influenza Epidemiological Surveillance Information System of Brazil
PC	Principal Component
MV	Mechanical ventilation
ICU	Intensive care unit
MCA	Multiple Correspondence Analysis
RR	Relative Risk
CI	Confidence Interval

## References

[1] Huang C., Wang Y., Liu X., Ren L., Zhao J., Hu Y., Zhang L., Fan G., Xu J. (2020). Clinical features of patients infected with 2019 novel coronavirus in Wuhan, China. Lancet.

[2] Brasil. Ministério da Saúde Orientações Sobre a Otimização do uso de Oxigênio e Suporte Ventilatório em Pacientes Graves com COVID-19. https://docs.bvsalud.org/biblioref/2021/05/1179870/orientacoes-sobre-otimizacao-do-uso-de-oxigenio-e-suporte-vent_DRIvHhs.pdf.

[3] Zhou Y., Chi J., Lv W., Wang Y. (2021). Obesity and diabetes as high-risk factors for severe coronavirus disease 2019 (COVID-19). Diabetes/Metabolism Res. Rev..

[4] Chen Y., Klein S.L., Garibaldi B.T., Li H., Wu C., Osevala N.M., Li T., Margolick J.B., Pawelec G., Leng S.X. (2021). Aging in COVID-19: Vulnerability, immunity and intervention. Ageing Res. Rev..

[5] Benski C., Di Filippo D., Taraschi G., Reich M.R. (2020). Guidelines for pregnancy management during the COVID-19 pandemic: A public health conundrum. Int. J. Environ. Res. Public Health.

[6] Souza A.S.R., Amorim M.M.R. (2021). Maternal mortality by COVID-19 in Brazil. Rev. Bras. Saúde Matern. Infant..

[7] Salem D., Katranji F., Bakdash T. (2021). COVID-19 infection in pregnant women: Review of maternal and fetal outcomes. Int. J. Gynecol. Obstet..

[8] Kumar R., Yeni C.M., Utami N.A., Masand R., Asrani R.K., Patel S.K., Kumar A., Yatoo M.I., Tiwari R., Natesan S. (2021). SARS-CoV-2 infection during pregnancy and pregnancy-related conditions: Concerns, challenges, management and mitigation strategies–a narrative review. J. Infect. Public Health.

[9] Allotey J., Stallings E., Bonet M., Yap M., Chatterjee S., Kew T., Debenham L., Llavall A.C., Dixit A., Zhou D. (2020). Clinical manifestations, risk factors, and maternal and perinatal outcomes of coronavirus disease 2019 in pregnancy: Living systematic review and meta-analysis. BMJ.

[10] Takemoto M.L., Menezes M.d.O., Andreucci C.B., Nakamura-Pereira M., Amorim M.M., Katz L., Knobel R. (2020). The tragedy of COVID-19 in Brazil: 124 maternal deaths and counting. Int. J. Gynecol. Obstet..

[11] Kotlar B., Gerson E., Petrillo S., Langer A., Tiemeier H. (2021). The impact of the COVID-19 pandemic on maternal and perinatal health: A scoping review. Reprod. Health.

[12] Brasil. Ministério da Saúde Guia de Vigilância Epidemiológica Emergência de Saúde Pública de Importância Nacional pela Doença pelo Coronavírus 2019. https://www.gov.br/saude/pt-br/centrais-de-conteudo/publicacoes/guias-e-manuais/2021/guia-de-vigilancia-epidemiologica-covid-19-3.pdf/view.

[13] Villar J., Ariff S., Gunier R.B., Thiruvengadam R., Rauch S., Kholin A., Roggero P., Prefumo F., Do Vale M.S., Cardona-Perez J.A. (2021). Maternal and neonatal morbidity and mortality among pregnant women with and without COVID-19 infection: The INTERCOVID multinational cohort study. JAMA Pediatr..

[14] Godoi A.P.N., Bernardes G.C.S., Almeida N.A.d., Melo S.N.d., Belo V.S., Nogueira L.S., Pinheiro M.d.B. (2021). Severe Acute Respiratory Syndrome by COVID-19 in pregnant and postpartum women. Rev. Bras. Saúde Matern. Infant..

[15] Francisco R.P.V., Lacerda L., Rodrigues A.S. (2021). Obstetric Observatory BRAZIL-COVID-19: 1031 maternal deaths because of COVID-19 and the unequal access to health care services. Clinics.

[16] Diniz D., Brito L., Rondon G. (2022). Maternal mortality and the lack of women-centered care in Brazil during COVID-19: Preliminary findings of a qualitative study. Lancet Reg. Health —Am..

[17] James G., Witten D., Hastie T., Tibshirani R. (2013). An Introduction to Statistical Learning.

[18] Li Z., Wang L., Huang L.s., Zhang M., Cai X., Xu F., Wu F., Li H., Huang W., Zhou Q. (2021). Efficient management strategy of COVID-19 patients based on cluster analysis and clinical decision tree classification. Sci. Rep..

[19] San-Cristobal R., Martín-Hernández R., Ramos-Lopez O., Martinez-Urbistondo D., Micó V., Colmenarejo G., Villares Fernandez P., Daimiel L., Martínez J.A. (2022). Longwise Cluster Analysis for the Prediction of COVID-19 Severity within 72 h of Admission: COVID-DATA-SAVE-LIFES Cohort. J. Clin. Med..

[20] Cui W., Cabrera M., Finkelstein J. (2020). Latent COVID-19 clusters in patients with chronic respiratory conditions. Integr. Citiz. Centered Digit. Health Soc. Care.

[21] Raposo L.M., Abreu G.F.D., de Medeiros Cardoso F.B., Alves A.T.J., Rosa P.T.C.R., Nobre F.F. (2022). Symptom-based clusters of hospitalized patients with severe acute respiratory illness by SARS-CoV-2 in Brazil. J. Infect. Public Health.

[22] Kenny G., McCann K., O’Brien C., Savinelli S., Tinago W., Yousif O., Lambert J.S., O’Broin C., Feeney E.R., De Barra E. (2022). Identification of distinct long COVID clinical phenotypes through cluster analysis of self-reported symptoms. Open Forum Infect Dis..

[23] Benham J.L., Atabati O., Oxoby R.J., Mourali M., Shaffer B., Sheikh H., Boucher J.C., Constantinescu C., Leigh J.P., Ivers N.M. (2021). COVID-19 vaccine–related attitudes and beliefs in Canada: National cross-sectional survey and cluster analysis. JMIR Public Health Surveill..

[24] Husson F., Lê S., Pagès J. (2011). Exploratory Multivariate Analysis by Example using R.

[25] Greenacre M.J. (1984). Theory and Applications of Correspondence Analysis.

[26] Backhaus K., Erichson B., Gensler S., Weiber R., Weiber T. (2021). Multivariate Analysis: An Application-Oriented Introduction.

[27] Johnson R.A., Wichern D.W. (1992). Applied Multivariate Statistical Analysis.

[28] Mirbeyk M., Saghazadeh A., Rezaei N. (2021). A systematic review of pregnant women with COVID-19 and their neonates. Arch. Gynecol. Obstet..

[29] Dashraath P., Wong J.L.J., Lim M.X.K., Lim L.M., Li S., Biswas A., Choolani M., Mattar C., Su L.L. (2020). Coronavirus disease 2019 (COVID-19) pandemic and pregnancy. Am. J. Obstet. Gynecol..

[30] Teixeira M.d.L.B., Costa Ferreira Júnior O.d., João E., Fuller T., Silva Esteves J., Mendes-Silva W., Carvalho Mocarzel C., Araújo Maia R., Theodoro Boullosa L., Gonçalves C.C.A. (2021). Maternal and neonatal outcomes of SARS-CoV-2 infection in a cohort of pregnant women with comorbid disorders. Viruses.

[31] Makvandi S., Ashtari S., Vahedian-Azimi A. (2020). Manifestations of COVID-19 in pregnant women with focus on gastrointestinal symptoms: A systematic review. Gastroenterol. Hepatol. Bed Bench.

[32] Mutiawati E., Fahriani M., Mamada S.S., Fajar J.K., Frediansyah A., Maliga H.A., Ilmawan M., Emran T.B., Ophinni Y., Ichsan I. (2021). Anosmia and dysgeusia in SARS-CoV-2 infection: Incidence and effects on COVID-19 severity and mortality, and the possible pathobiology mechanisms-a systematic review and meta-analysis. F1000Research.

[33] Takemoto M.L., Menezes M.d.O., Andreucci C.B., Knobel R., Sousa L., Katz L., Fonseca E.B., Nakamura-Pereira M., Magalhães C.G., Diniz C.S. (2020). Clinical characteristics and risk factors for mortality in obstetric patients with severe COVID-19 in Brazil: A surveillance database analysis. BJOG Int. J. Obstet. Gynaecol..

[34] Menezes M.O., Takemoto M.L., Nakamura-Pereira M., Katz L., Amorim M.M., Salgado H.O., Melo A., Diniz C.S., de Sousa L.A., Magalhaes C.G. (2020). Risk factors for adverse outcomes among pregnant and postpartum women with acute respiratory distress syndrome due to COVID-19 in Brazil. Int. J. Gynecol. Obstet..

[35] Lassi Z.S., Ali A., Das J.K., Salam R.A., Padhani Z.A., Irfan O., Bhutta Z.A. (2021). A systematic review and meta-analysis of data on pregnant women with confirmed COVID-19: Clinical presentation, and pregnancy and perinatal outcomes based on COVID-19 severity. J. Glob. Health.

[36] Overtoom E.M., Rosman A.N., Zwart J.J., Vogelvang T.E., Schaap T.P., van den Akker T., Bloemenkamp K.W. (2022). SARS-CoV-2 infection in pregnancy during the first wave of COVID-19 in the Netherlands: A prospective nationwide population-based cohort study (NethOSS). BJOG Int. J. Obstet. Gynaecol..

[37] La Verde M., Riemma G., Torella M., Cianci S., Savoia F., Licciardi F., Scida S., Morlando M., Colacurci N., De Franciscis P. (2021). Maternal death related to COVID-19: A systematic review and meta-analysis focused on maternal co-morbidities and clinical characteristics. Int. J. Gynecol. Obstet..

[38] Brislane Á., Larkin F., Jones H., Davenport M.H. (2021). Access to and quality of healthcare for pregnant and postpartum women during the COVID-19 pandemic. Front. Glob. Women’S Health.

[39] Wastnedge E.A., Reynolds R.M., Van Boeckel S.R., Stock S.J., Denison F.C., Maybin J.A., Critchley H.O. (2021). Pregnancy and COVID-19. Physiol. Rev..

[40] Scheler C.A., Discacciati M.G., Vale D.B., Lajos G.J., Surita F., Teixeira J.C. (2021). Mortality in pregnancy and the postpartum period in women with severe acute respiratory distress syndrome related to COVID-19 in Brazil, 2020. Int. J. Gynecol. Obstet..

[41] Chmielewska B., Barratt I., Townsend R., Kalafat E., van der Meulen J., Gurol-Urganci I., O’Brien P., Morris E., Draycott T., Thangaratinam S. (2021). Effects of the COVID-19 pandemic on maternal and perinatal outcomes: A systematic review and meta-analysis. Lancet Glob. Health.

