# Supplementary material for: Clusters of Pregnant Women with Severe Acute Respiratory Syndrome Due to COVID-19: An Unsupervised Learning Approach
Source: Int J Environ Res Public Health. 2022 Oct 19;19(20):13522. doi: 10.3390/ijerph192013522 (PMC9603349; doi:10.3390/ijerph192013522)
Supplement: Supplementary file 1 [file ijerph-19-13522-s001.zip › ijerph-1920167-supplementary/Figure S1.pdf]

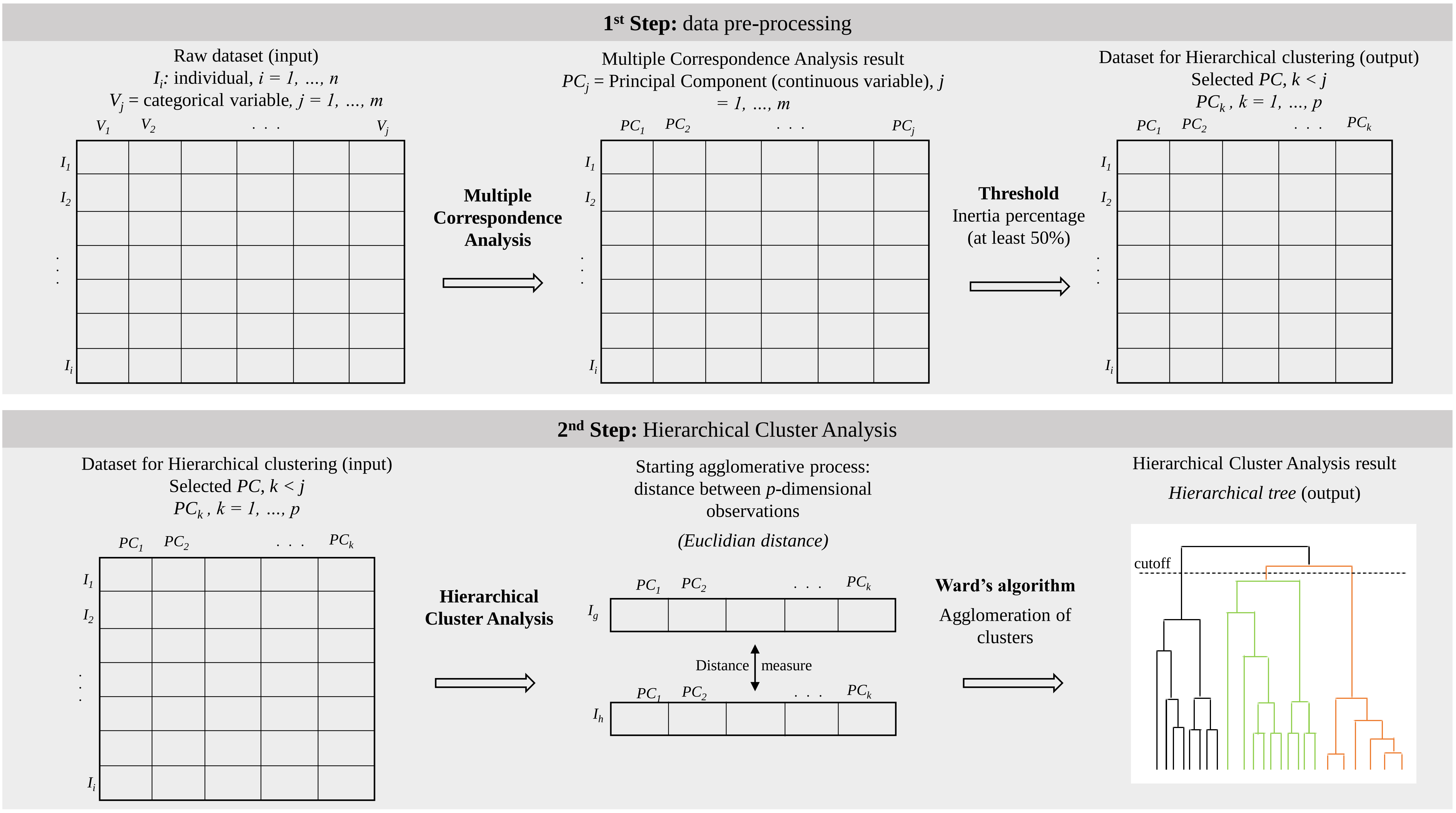
1st Step: data pre-processing
Raw dataset (input)
I_i: individual, i = 1, …, n
V_j = categorical variable, j = 1, …, m
V_1 V_2 . . . V_j
I_1
I_2
I_i
Multiple Correspondence Analysis
Multiple Correspondence Analysis result
PC_j = Principal Component (continuous variable), j = 1, …, m
PC_1 PC_2 . . . PC_j
Threshold
Inertia percentage (at least 50%)
Dataset for Hierarchical clustering (output)
Selected PC, k < j
PC_k , k = 1, …, p
PC_1 PC_2 . . . PC_k
2nd Step: Hierarchical Cluster Analysis
Dataset for Hierarchical clustering (input)
Selected PC, k < j
PC_k , k = 1, …, p
Hierarchical Cluster Analysis
Starting agglomerative process: distance between p-dimensional observations
(Euclidian distance)
I_g
I_h
Distance measure
Ward's algorithm
Agglomeration of clusters
Hierarchical Cluster Analysis result
Hierarchical tree (output)
cutoff
