# Supplementary figures and images for: Clusters of Pregnant Women with Severe Acute Respiratory Syndrome Due to COVID-19: An Unsupervised Learning Approach
Source: Int J Environ Res Public Health. 2022 Oct 19;19(20):13522. doi: 10.3390/ijerph192013522 (PMC9603349; doi:10.3390/ijerph192013522)

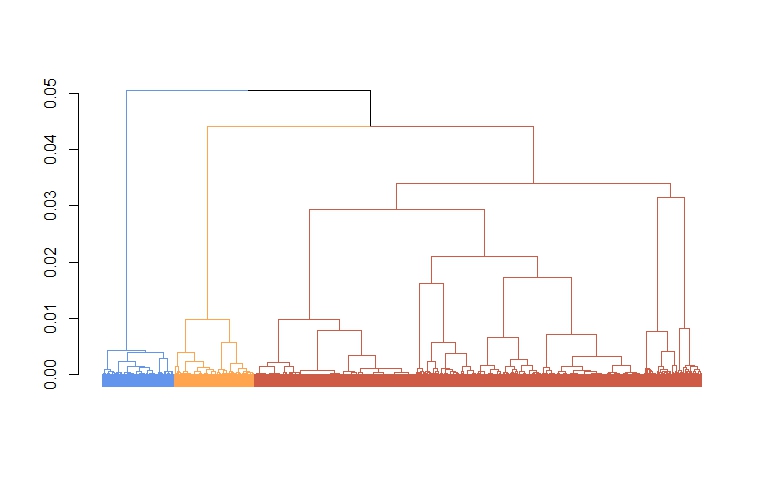

Supplement: Supplementary file 1 [file ijerph-19-13522-s001.zip › ijerph-1920167-supplementary/Figure S2.jpeg]

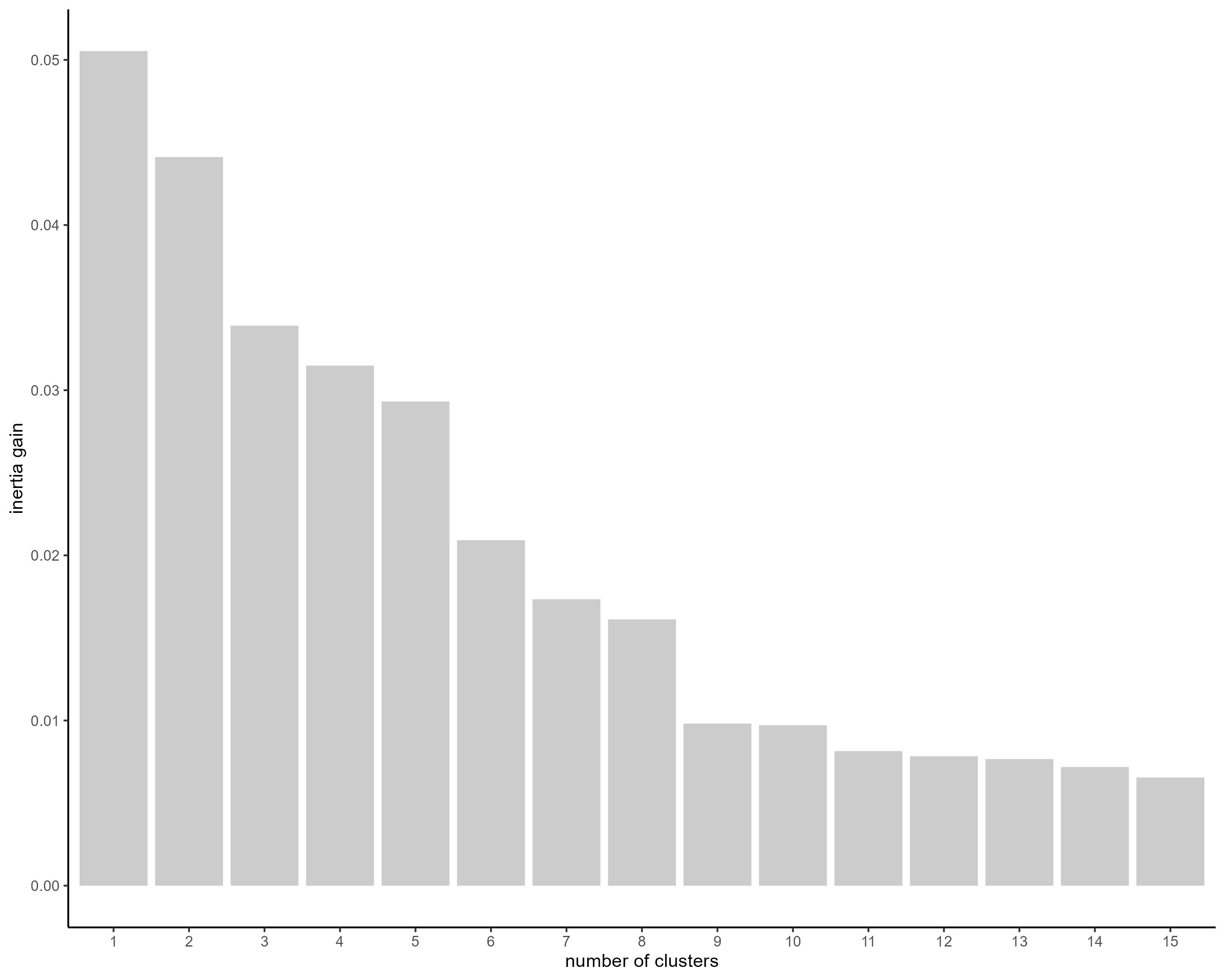

Supplement: Supplementary file 1 [file ijerph-19-13522-s001.zip › ijerph-1920167-supplementary/Figure S3.jpeg]
